# Inequality, chronic undernutrition, maternity, and diabetes mellitus as the determinant of anemia among ever-married women in Bangladesh

**DOI:** 10.1186/s12889-021-10362-2

**Published:** 2021-02-06

**Authors:** G. M. Rabiul Islam

**Affiliations:** Department of Food Engineering and Tea Technology, Shahjalal University of Science and Technology, Sylhet, 3114 Bangladesh

**Keywords:** Anemia, Reproductive age group, Inequality, Diabetes, Maternity, BMI

## Abstract

**Background:**

Anemia is a public health concern at a global level*.* This study aims to delineate the association between household economic inequalities, and anemia among reproductive age group women in Bangladesh, along with other confounders.

**Methods:**

A cross-sectional population sample from Bangladesh Demographic and health survey data comprising of 5920 reproductive age group women aged between 15 and 49 years was used in this study. The analyses were performed through the application of proportional odds into four models (viz., Model:1 socio-economic, Model:2 socio-demographic factors, Model 3: diabetics and maternity statis, Model 4: nutritional status.)

**Results:**

The respondents from low and medium socioeconomic status (SES) households vs. richest households were 1.90 (95% CI, 1.65,2.17; *p* < .01) and 1.41 (95% CI, 1.12,1.78; *p* < .01) times more likely to suffer from anemia (Model 1). From the model 2 it appears that he likelihood of being anemic reduces for the low and medium SES groups vs. high SES group when sociodemographic variables are added (OR, 1.69, 95% CI, 1.43,1.99 and OR, 1.35, 95% CI, .07,1.70; *p* < 0.01, respectively). Model 3 evident that after adding the variables of diabetes and maternity status, the association between having anemia belonging to low and medium SES vs. high SES becomes weaker (OR1.36, 95% CI,0.85,2.15 and OR, 1.15, 95% CI, 0.6,2.19; *p* > .05, respectively). Moreover, the strength of the association between anemia and the subjects with pregnant vs. the subjects without these (OR: 1.7 (1.12, 4.02; *p < 0.05*) 1.47(0.11,3.399; p < 0.05) conditions was reduced after factoring body mass index (BMI, model 4). Besides, thin women (MBI < 18.50 Kg/m^2^) shows significantly become more likely to be anemic in comparison to women of normal BMI (OR:1.34, .92,1.96; *p < 0.05*) (model 4).

**Conclusions:**

BMI, pregnancy, and diabetes mellitus were observed to be significantly associated with anemia, and the strength of the association was low with anemia and socioeconomic inequality.

## Background

Despite significant advances in the field of medicine and healthcare, anemia continues to be a major public health problem worldwide [1]. Individuals from low- and middle-income countries are particularly affected [2]. In 2011, the World Health Organization (WHO) estimated that globally half a billion women are affected with anemia, among them 29% are non-pregnant, and 38% are pregnant women, and the prevalence of anemia was the highest in south Asia and central and west Africa [3]. Between 1990 and 2010, increasing efforts were made to combat anemia but the global burden of the disease still remains surprisingly substantial [1]. Women of reproductive age are especially vulnerable to the disease, and a 50% reduction is the second global nutrition target for 2025, set forth by the World Health Assembly [4].

Anemia during pregnancy is a risk factor for preterm delivery and subsequent low birth weight, and possibly for inferior neonatal health [5, 6]. The high prevalence of anemia is of great concern because it is associated with cognitive, motor, and socio-emotional abilities [7]. These abilities are linked with academic achievement and economic productivity [8]. Anemia is a result of a wide variety of causes that are typically known to coexist. There is a close association with diabetes mellitus and anemia as well as pregnancy [9]. Besides, in Bangladesh, the prevalence of diabetic patient is increasing [10]. Other factors contributing to anemia include iron deficiency; other micronutrient deficiencies such as folate, vitamin B12, and vitamin-A; diabetes; chronic kidney disease (CKD); parity; acute and chronic infections; and chronic undernutrition [11, 12]. Socioeconomic factors have also been associated with anemia as food insecurity, inadequate water, sanitation and hygiene have been shown to increase the risk of infectious diseases and decreasing the availability and consumption of micronutrient-rich foods [13, 14]. In Bangladesh, analysis of the national representative data recently released by the Demographic and Health Surveys (BHDS) 2011, accurately summarizes the magnitude of the problem among women of reproductive age. In the BDHS 2011 sample, the prevalence of anemia among reproductive age group women between the ages of 15 and 49 years was approximately 42%. Thirty-six percent of these women were classified as mildly anemic (hemoglobin < 110–119.99 g/L), 7% as moderately anemic (hemoglobin < 80–109.99 g/L), and 1% as severely anemic (hemoglobin < 80 g/L) [15]. Clearly, anemia in women of reproductive age is of public health concern and is a significant contributor to comorbidities in Bangladesh. Given the multifactorial nature of this disease, anemia prevention and treatment require an integrated approach. Therefore, it is imperative to identify the factors associated with anemia in order to effectively combat the disease.

The association between socioeconomic status and anemia has been studies in Southeast Ethiopia, Northeast Ethiopia, Andhra Pradesh India, Mexico Peru, and Egypt demonstrated that women in poorer households tend to be more anemic and undernourished as compared to those in better households [16–19]. However, despite the heavy burden of anemia related comorbidity in Bangladesh, no epidemiologic studies have been conducted in order to elucidate the risk factors associated with anemia emphasizing inequality among women of reproductive age in Bangladesh. Though, Kamruzzaman et al., 2016 analyzed anemia and its contributing factor in Bangladesh but did not emphasize wealth- or income-related inequality. Therefore, the aim of this study was to find what variables would show a strong association with varying severity of anemia in order “to contribute to policymaking and be instrumental in the programs designed to prevent anemia in Bangladeshi women.

## Methods

### Study settings

We used data from Bangladesh Demographic and Health Surveys (DHS) collected by ICF International, which were conducted from July 8 to December 27, 2011 in collaboration with the National Institute of Population Research and Training (NIPORT) of the Ministry of Health and Family Welfare of Bangladesh, and Mitra and Associates, a Bangladeshi research firm [15].

Bangladesh has seven administrative divisions: Barisal, Chittagong, Dhaka, Khulna, Rajshahi, Rangpur, and Sylhet. Each division is subdivided into district, and each district into sub-district. Thus, the divisions allow the country as a whole to be easily separated into rural and urban areas. The sampling in BDHS was done through a stratified, multistage cluster selection approach. In the first stage, 600 enumeration areas (EAs), of which 207 were from urban areas and 393 were from rural areas, were selected. This primary sampling EAs were selected from the sampling frame created for the 2001 Bangladeshi census. In the second stage, a systematic sample of 30 households on average was selected per EA to provide statistically reliable estimates of key demographic and health variables for the country, for urban and rural areas separately and for each of the seven divisions of the country. Next, a total of 1800 selected households, interviews were successfully completed in 17,141 households. Finally, within these households, a total of 18,222 reproductive age group were successfully interviewed. A total of 5920 reproductive age group women aged between 15 and 49 years who resided in one-third of the randomly selected households were selected as the subjects for the biomarker assay for anemia study. After excluding the subjects for whom associated hemoglobin data were missing, a total of 5886 women were selected for the final analysis.

### Outcome

In this study, hemoglobin concentration was used to diagnose anemia. The Hemocue system was used to estimate the concentration of hemoglobin in the capillary blood. The prevalence was adjusted for individual sample weight [20], altitude and smoking status using the formula suggested by the Center for Disease Prevention of United State [15] adopted by WHO [21] and BDHS [22], as hemoglobin concentrations differ substantially depending on the altitude and smoking status. For the purpose of this study, “anemia” was defined as per the 2011 WHO’s guidelines for the diagnosis of anemia; the same definition was also adopted by the Demographic Health Surveys [21, 22]. Briefly, in the case of pregnant women, a hemoglobin concentration of less than 70 g/L was used to define severe anemia, between 70 and 99.99 g/L was used for defining moderate anemia while 100–109.99 g/L was taken to correspond to mild anemia. In non-pregnant women, a hemoglobin concentration of less than 80 g/L was used to define severe anemia, 80–109.99 g/L for moderate anemia, and 110–119.99 g/L to correspond to mild anemia. However, as only 0.14% of women were classified as being severely anemic, for the purposes of the regression modeling, the severe and moderate groups were combined to create a severe/moderate category (hemoglobin concentration of < 100 g/L in pregnant women and less than 110 g/L in non-pregnant women) to avoid the problems of zero cell counts in the models.

### Explanatory variables

Socioeconomic status, defined as the wealth status of the individual, was used as a key predictor in this study. The wealth index, used in the BDHS survey, has been used in many previous DHS and other country-level surveys to measure the inequalities in a variety of confounders such as household characteristics, health, and other services, and in health outcomes [15]. This serves as an indicator of household wealth level that is found to be consistent with both expenditures as well as income measures [23]. The BDHS calculated the index using household asset data that comprises information on the availability of electricity, housing facilities, possession of household durable good and ownership of a homestead and land via principal components analysis. In this study, the wealth index score of the 2011 BDHS survey was used as a proxy for SES and inequality was measured by dividing the wealth index into tertiles and categorizing them as “low” “medium” and “high”. This study also included other related explanatory variables, such as respondent education level (none/primary school/secondary school or higher), place of residence (rural/urban), employment status (employed/unemployed) sex of households head, number of child, menopausal age, family size, maternity and nutritional status (BMI), etc. (see details in Table 1).

**Table 1:** The variables tested for the association with anemia in the logistic regression Models 1–4

Model 1:	Socio-economic status	Household-level wealth index (Low, Medium or High)
Model 2:	Model 1 variable and Sociodemographic variables	
	Respondent Highest Education Level:	None/completed primary/completed secondary or higher
	Place of residence:	Rural/Urban
	Employment status:	Employed/Not employed
	Sex of house old head:	Male/Female
	Number of children:	None/One/Two/Three or more
	^a^women reproductive age:	Early reproductive (< 20 years)/Middle reproductive (20–38 years) /Late reproductive (39–42 years)/Early premenopause (43–46 years)/Late premenopause (< 46 years)
	Family size:	Small/Large (Number of family members 7 or over which is based on mean number of members in a household).
Model 3:	Model 2 variables and diabetes and maternity status	
	Maternity status:	Pregnant/Lactating/Neither
	^b^Fasting plasma glucose levels:	Normal/Peri diabetic/Diabetes
Model 4:	Model 3 variables and BMI	
		Underweight/thin (< 18.50 kg/m^2^); Normal (18.50, 24.99 kg/m^2^); Overweight (25,30 kg/m^2^); Obese (> 30 kg/m^2^)

^a^ The menopausal age is considered from the study of Bharadwaj et al. [24]

^b^ The 2011 BDHS measured the blood glucose level using the HemoCue 201+ blood glucose analyzer (Teleflex Medical L.P., Markham, Canada) in whole blood obtained by finger prick from the capillaries in the middle or ring finger after an overnight fasting—an approach that is widely used in resource-limited settings. The definition of “diabetes” is adopted from the Demographic Health Surveys report, that is, fasting plasma glucose greater than or equal to 7.0 mmol/L or use of anti-diabetic medication. Subjects with “pre-diabetes” were defined as subjects with fasting plasma glucose concentrations in the range of 6.1–6.9 mmol/L and not taking anti-diabetic medication [25]. For this classification of “diabetes” and “prediabetes”, the cutoff point for fasting plasma glucose was according to the recommendation of the American Diabetes Association [26] and International Diabetes Federation [27]

### Ethical considerations

The data collection procedure for the BDHS was carried out with the approval of the ORC Macro-institutional review board (ICF Macro Project Number: 631561.0.000.00.091.01), and National Research Ethics Committee, Bangladesh Medical Research Council (BMRC/NREC/2010–2013/537). As per the guidelines of the BDHS, prior to the interview, informed consent was obtained from individual respondents and this was followed by an oral explanation given by the interviewers. The respondents were informed of the voluntary nature of the survey, the potential risks involved in participation, the purpose of gathering the data (assessment of health needs and planning health services) and the confidentiality of the results of the individual interview along with the free of charge nature of the examination.

### Statistical analysis

Pearson’s chi-square test was used to determine statistically significant differences between the categories of the CDC/WHO hemoglobin grouping variable in relation to other explanatory variables. We also used the concentration indices to quantify the degree of socioeconomic related inequality of being anemic. Concentration indices are frequently used to measure inequality in one variable over the distribution of another. Most commonly, they are applied to the measurement of socioeconomic-related inequality in health [28]. Therefore, we also calculated the concentration index of being underweight, diabetes, and pre-diabetes across the SES as variables also consider as an independent confounder.

Ordered logit using maximum likelihood estimation was used to identify the socioeconomic and other determinants of anemia status. To account for the complex survey design, this study included strata (i.e.,7 division of Bangladesh) and clustering variable (primary sampling unit) by using the survey option in Stata. Four models were tested in the ordered logit models to identify the consequence of the different variables associated with anemia (Table 1). Model 1 included SES, a variable that indicated the level of household wealth. This allowed this study to assess whether different SES groups exhibit differences in the likelihood of being anemic. In Model 2, sociodemographic variables were introduced in addition to socioeconomic status. Two additional models were also tested considering the variables already included in the earlier models. Model 3 introduced diabetes and maternity as factors while Model 4 used BMI. The variables are detailed in Table 1. The models allowed for an in-depth analysis of the significance of SES as correlated with anemia status while controlling for a range of other factors. In addition, this methodology allowed for the identification of factors that reduced the significance of SES in each model thus enabling the identification of variables associated with SES as well as hemoglobin concentrations in women. Analyses were performed with Stata (version 13; Stata Corp LP).

## Results

### Descriptive analysis

Table 2 summarizes the proportion of women in each anemia group, when the groups were classified using the explanatory variables and the chi-square test. The chi-square test indicated that the differences observed in the percentage of women classified as anemic based on factors such as SES, respondent education level, place of residence, age, maternity status, fasting blood plasma glucose level and nutritional status, were statically significant (*p* < 0.05). The concentration index indicates that the trends of anemia, underweight, prediabetes and diabetes were stronger in the individuals belonging to low SES category (Table 3).

**Table 2:** Percentages of women with anemia by standard of explanatory variables (*N* = 5886)

		Anemia status by hemoglobin level%				
Variable	n (%)	Not anemic	Mild	Moderate/ Sever	Chi-square result	*P* value
**Socio-economic status**						
Low	4265 (72.46)	54.59	37.50	7.90	92.63	<.01
Medium	434 (7.37)	59.38	37.05	3.57		
High	1187 (20.17)	70.94	24.00	5.06		
**Socio-demographic factors**						
Highest Education level						
No education	2605 (44.26)	54.46	37.00	8.54	46.88	<.01
Completed primary	2553 (43.37)	59.78	34.23	5.98		
Completed secondary or higher	728 (12.37)	67.57	27.67	4.76		
**Place of residence**						
Rural	3863 (65,63)	55.92	36.81	7.27	26.11	<.01
Urban	2023 (34.37)	62.91	30.50	6.52		
**Employment status**						
Employed	853 (85.49)	58.35	34.62	7.04	0.50	.80
Not employed	5027 (14.51)	57.33	35.68	6.99		
**Sex of house old head**						
Male	5301 (90.06)	57.95	34.86	7.18	1.93	.38
Female	585 (9.94)	61.12	33.52	5.36		
**Number of child**						
No child	2903 (39.10	58.09	35.01	6.90	2.49	.87
One	2145 (36.44)	57.61	35.01	7.39		
Two	670 (11.38)	60.87	32.34	6.80		
Three or more	168 (2.85)	58.20	36.38	5.42		
**Age**						
Early reproductive	889 (15.10)	57.13	35.18	7.69	16.13	.04
Middle reproductive	3502 (59.50)	58.68	34.65	6.67		
Late reproductive	637 (10.82)	62.70	31.21	6.09		
Early premenopause	488 (8.29)	52.78	34.21	9.01		
Late premenopause	370 (6.29)	56.01	36.20	7.79		
**Family size**						
Small	4835 (82.14)	57.93	34.77	7.30	3.24	.19
Large	1051 (17.86)	69.60	34.62	5.78		
**Maternity status**						
Pregnant	372 (6.32)	47.92	38.20	13.88	83.17	.01
Lactating	1356 (23.06)	58.10	34.11	7.79		
Neither	4158 (70.06)	61.61	32.29	6.10		
Diabetes						
Normal	5076 (86.25)	59.67	33.59	6.74	1.26	.04
Prediabetic	523 (8.87)	60.89	35.10	4.01		
Diabetic	287 (4.86)	59.95	32.70	7.36		
BMI^**^						
Underweight (< 18.50)	1395 (23.86)	49.58	42.26	8.15	94.68	<.01
Normal (18.50, 24.99)	3422 (58.20)	58.44	34.22	7.35		
Overweight (25,30)	886 (14.81)	68.91	26.22	4.87		
Obese (> 30)	183 (3.13)	72.70	34.67	2.63		

^**^Weight gain was adjusted incase of pregnancy to calculate the BMI [29], that also adopt by BDHS [15]

**Table 3:** Summary measures of SES inequality and the prevalence of anemia, underweight, diabetes and prediabetes among reproductive age group women aged 15 to 49 years

Variable	Concentration index^c^	Standard error (SE)	*P*-value
Underweight women	−.52	.03	<.001
Anemic women	−.42	.01	.002
Pre-diabetic women	−.32	.01	.04
Diabetic women	−.30	.04	.02

^C^ Obtained by the formula *2σ*^*2*^_*r*_ *(h*_*i*_*/μ) = α + βr*_*i +*_*ε*_*i;,*_ *h* is the health variable whose inequality is being measured, *μ* is its mean, *r*_*i*_ is the *i*th individual’s fractional rank (for example if a women is underweight and which SES group she is belonging) in the socioeconomic distribution, *σ*^*2*^_*r*_ is the variance of the fractional rank, The estimate *β* is equal to the concentration index, *r*_*i =*_ underweight women, anemic women, pre-diabetic women, diabetic women α is constant (underweight women = .65, anemic women = .95, pre-diabetic women = .94, diabetic women = .84) and *ε*_*i*_ is the error term. Concentration index has a negative value when the health indicator is concentrated among the disadvantaged; A positive value when the health indicator is concentrated among the advantaged; When there is no inequality the value is equals 0; the theoretical maximum is ±1

### Effect of household economic inequality and other confounders

The results of the ordered logit models are presented in Table 4. The women from low and medium SES groups showed the higher odds (OR, 1.90, 95% CI, 1.65–2.17; *p* < .01 and OR, 1.41, 95% CI, 1.12–1.78; *p* < .01) of being anemic when compared to the high SES group in an unadjusted model (Model 1). From Model 2, it is evident that women with no education or primary educational attainment were more likely to be anemic in comparison to those who had completed secondary or higher education (OR, 1.22, 95% CI, 1.05–1.42 & OR, 1.15 95% CI, 1.01–1.3; *p* < 0.01, respectively).

**Table 4:** The determinants of anemia in women in Bangladesh (obtained from ordinal logistic regression models) (N = 5886)

	Model 1		Model 2		Model 3		Model 4	
	Odds ratio (95% CI)	P Value	Odds ratio (95% CI)	P Value	Odds ratio (95% CI)	P Value	Odds ratio (95% CI)	P Value
**Socioeconomic status**								
Low	1.90 (1.65,2.17)	< 0.01	1.69 (1.43,1.99)	< 0.01	1.36 (0.85,2.15)	0.20	1.23 (0.69, 1.83)	0.62
Medium	1.41 (1.12,1.78)	< 0.01	1.35 (1.07,1.70)	< 0.01	1.15 (0.6,2.19)	0.66	1.11 (0.58, 2.11)	0.76
High	1		1		1		1	
Socio-demographic factors								
**Education**								
No education			1.22 (1.05,1.42)	< 0.01	1.21 (0.79,1.84)	0.37	1.17 (.76, .80)	0.46
Completed primary			1.15 (1.01, 1.31)	0.03	1.25 (0.82,1.91)	0.30	1.29 (.8,1.88)	0.35
Completed secondary or higher			1		1			
**Place of residence**								
Rural			1.07 (0.94,1.22)	0.30	1.06 (0.72,1.54)	0.78	.99 (67,1.46)	0.96
Urban			1		1		1	
**Currently working**								
Yes			1.05 (.90,1.23)	0.49	1.08 (0.68,1.53)	0.94	0.99 (.67,1.46)	0.98
No			1				1	
**Sex of house old head**								
Female			.93 (0.78,1.12)	0.45	.79 (0.50,1.27)	0.34	.82 (0.65,1.50)	0.41
Male			1		1		1	
**Number of child**								
No child			1		1		1	
One			0.99 (0.89,1.12	0.96	0.96 (0.69,1.33)	0.81	0.95 (0.69,1.32)	0.77
Two			0.90 (0.75,1.07)	0.24	1.21 (0.73,2.01)	0.45	1.23 (0.74,2.06)	0.42
Three or more			1.01 (0.73,1.40)	0.94	1.08 (0.39,2.97)	0.88	1.06 (0.38,2.92)	0.91
**Age**								
Early reproductive			1		1		1	
Middle reproductive			0.92 (0.78,1.07)	0.26	0.77 (0.49,1.22)	0.27	.83 (0.52,1.32)	0.44
Late reproductive			0.79 (0.64,0.99)	0.04	0.59^**^(0.37,0.93)	0.02	.62 (0.39,0.98)	0.04
Early premenopause			1.12 (0.88,1.42)	0.35	0.89 (0.55,1.44)	0.63	.94 (0.58,1.53)	0.81
Late premenopause			1.10 (0.86,1.43)	0.43	0.76 (0.82,1.48)	0.53	.71 (0.73, 1.36)	0.17
**Family size**								
Small			1		1	1	1	
Large			1.01 (0.87,1.16)	0.91	1.05 (0.69,1.59)	1.05	1.06 (.70,1.61)	0.79
**Diabetics and Maternity status**								
**Maternity status**								
Pregnant					1.74 (1.12, 4.20)	< 0.01	1.7 (1.12,4.02)	0.03
Lactating					1.62 (1.09,3.70)	0.03	1.47 (0 .11,3.99)	0.14
Neither					1		1	
**Fasting plasma glucose levels**								
Normal					1		1	
Prediabetic					.67 (0.39,1.15)	0.15	.68 (0.39, 1.70)	0.17
Diabetic					1.67 (0.78,3.49)	0.18	1.74 (1.02, 3.68)	0.04
**Nutritional status**								
BMI								
Underweight (< 18.50)							1.34 (.92,1.96)	0.04
Normal (18.50, 24.99)							1	
Overweight (25,30)							.62 (.39,0.97)	0.13
Obese (> 30)							.66 (.30,1.45)	0.30

The likelihood of being anemic appear as slightly lowered for low and medium SES groups (model 2) in comparison to the high SES group when sociodemographic variables were added to the bivariate analysis (Model 1) (OR, 1.9, 95% CI, 1.65–2.17 to OR, 1.69, 95% CI, 1.43–1.99 and OR, 1.41, 95% CI, 1.21–1.78 to OR,1.35, 95% CI,1.07–1.7; *p* < 0.01, respectively).

After adding the variables for maternity and diabetes the likelihood of having anemia in the subjects from low and medium SES households vs. high SES households and individuals having primary and secondary or higher secondary education vs. their counterpart with no educational attainment became insignificant *(p* > 0.05). The maternity status was also found to be a significant factor contributing to anemia in Model 4. The pregnant women were observed to be approximately two times more prone to being mild anemic (OR, 1.70, 95% CI, 1.12–4.02; *p* < .05) while lactating women appeared to be insignificantly (OR, 1.47, 95% CI, 0.11–3.99; *p* > 0.05) anemic as compared to women who were neither pregnant nor lactating. Diabetes mellitus was found as an associate factor of anemia in the full model (Model 4). Women who were suffering from diabetes mellitus were observed to have 74% higher chance of being 1.74 times, (OR, 1.74, 95% CI 1.02–3.86; *p* < 0.05) for being mildly, moderately or severely anemic as compared to the subjects with normal blood plasma glucose levels. The overweight women with BMI in the range of 25–30 kg/m^2^ were observed to be approximately two times less likely to be anemic than those with a normal BMI (OR, 0.62, 95% CI, 0.39–0.97; *p* < .05). Similarly, the women belonging to the early reproductive (< 20 years) age group were observed to be around 38% times less likely to be anemic than those who belonged to the late reproductive age group (OR, .62, 95% CI, .39–.98; *p* < .05) (Model 4).

It was evident from Models 3 and 4 that after controlling the variable for nutritional status (BMI), the strength of the associations between anemia and pregnancy status became low (OR, 1.74, 95% CI, 1.12–4.20; *p* < 0.01 to OR, 1.7, 95% CI, 1.12–4.20; *p* < 0.05 vs. subject neither pregnant or lactating).

## Discussion

### Effect of household socioeconomic status on anemia

The results of this study indicated that the hypothesis correlating SES and anemia was partially supported (Model 1 and 2). In developing countries, it is well established that the people belonging to low SES groups suffer from problems such as poor housing, malnutrition, overcrowding, pollution, and increased exposure to infectious diseases [30, 31]. In comparison to high SES women, the women of the lower SES group were at a higher risk for anemia; this can be attributed to the factors such as the lack of access to the subject belonging to low SES own income or resources which in turn results in the lack of decision-making power and autonomy regarding issues such as free movement for treatment, antenatal care, medical facilities, education, etc. In addition, women belonging to the low SES category may also experience higher rates of infection as a result of poor sanitation, gynecological morbidity, and sexually transmitted diseases [32].

### Inference of demographics, diabetes, maternity, and BMI

The results evidence the fact that upon factoring in sociodemographic parameters with Model 1, the likelihood of the members of the low and medium SES group being anemic decreased as compared to the higher SES group. Among the sociodemographic factors analyzed in this study, the respondents with no education or those with only primary education had a higher chance of being anemic as compared to the women with higher educational attainment. These results are in parallel with a similar investigation conducted by Bentley and Griffiths for the National Family Health Survey 1998/99 (NFHS–2) wherein the authors discerned a similarity in the prevalence and determinants of anemia in the women in Andhra Pradesh, India [16]. In their study, Bentley and Griffiths demonstrated that in Andhra Pradesh, adding the variable of maternal education causes a reduction in the odds of being anemic for urban and rural women belonging to the medium and high SES groups as compared to high urban women [16].

The analysis of the results obtained from Model 3 indicates that the addition of diabetics and maternity status reduces the strength of association between having anemia with SES and the education level. This observation can be attributed to the fact that in Bangladesh fasting plasma glucose levels and maternity status are more prominent factors related to anemia as compared to SES and education levels. The precise relationship between diabetics and anemia could not be elucidated in this study. Several previously published studies have indicated that diabetes does not directly cause anemia, but certain complications associated with diabetes can contribute to the phenomenon. For example, both diabetes-related kidney disease (nephropathy) and nerve damage (neuropathy) can be instrumental to the development of anemia [33, 34]. Moreover, individuals suffering from diabetes can also have anemia as a result of not eating well or of having a condition that interferes with the proper absorption of nutrients.

The results of this study strongly indicate that women who are pregnant are more likely to be anemic as compared to women who are neither lactating nor pregnant (Models 3). Interestingly, as is evident from Models 3 and 4, the significance of the association between pregnancy reduces after controlling BMI. In developing countries, the prevalence of anemia during pregnancy is a well-recognized phenomenon [35, 36]. The major causative factors implicated in the emergence of anemia during pregnancy are iron-deficiency, folate-deficiency, and vitamin B12 deficiency [37–39]. During pregnancy, the body produces an increased amount of blood in order to support the growth and nutrient requirements of the fetus developing inside the mother’s womb. If pregnant women do not get enough iron or certain other nutrients, the body might not be able to produce enough red blood cells to constitute the additional blood. While it is normal to have mild anemia during pregnancy, even after adjusting the hemoglobin level for pregnancy as per WHO guidelines, 52.08% of pregnant women in Bangladesh are suffering from anemia, of which 13.88% fall in the moderately or severely anemic category (Table 1). Furthermore, anemia from iron deficiency is common in lactating women especially following the anemia in pregnancy [40, 41].

There is a lower risk of anemia associated with late reproductive age (Models 2–4) as compared to early reproductive age. This can be explained as a result of the lower frequency of pregnancy during this stage and early marriage in Bangladesh that increases a higher rate of pregnancy in early reproductive age [42, 43]. The addition of the nutritional status variable (BMI) in Model 4 reduces the likelihoods of anemia in the pregnant women in comparison to their counterpart neither pregnant nor lactating. The results of this study revealed that thin women (BMI < 18.5 kg/m^2^) were marginally more likely to be anemic as compared to women of normal BMI (Table 4). It is well known that being underweight is a risk factor for anemia especially if the low BMI is caused by insufficient diet [44]. Hence, improving the nutritional status (BMI) in thin women within the study population may have the potential to combat the situation.

Several studies from developing countries have documented that low SES is the main factor in being underweight [45–47]. Therefore, it can be postulated that in order to improve the BMI in thin women in Bangladesh, greater emphasis should be given to improve SES as well as education. Although this study reveals that being classified as normal or overweight is somewhat protective, more than 42% (see Table 2) of women with a normal BMI (18.50–24.99 kg/m^2^) were moderately or severely anemic. Additionally, more than 30% of overweight (BMI 25–30 kg/m^2^) women (Table 2) were also victim of some type of anemic disorders suggesting that this subgroup might be influenced by factors other than obvious resource constraints [48].

This study included certain special features that deserve to be mentioned, such as (a) the prevalence of anaemia was adjusted for altitude and smoking using the CDC formula adopted by WHO [21, 49]; (b) the order logit model was used to increase the robustness of estimates as well as to account for complex survey design strata, while the clustering variables were used for fitting the model for statistical analysis; (c) BDHS ensured that the interviewers were trained extensively and that the standardized measurement tools and techniques were used in the study. Despite the aforementioned strengths, this study also had some limitations. For example, this study did not include lifestyle parameters of the test subjects, including diet variables and other biomarkers of nutritional deficiencies such as folate, vitamin B12, and vitamin-A deficiency, all of which may impact the hemoglobin concentration. Furthermore, the information on acute and chronic inflammation, parasitic infections, and inherited or acquired disorders that affect hemoglobin synthesis were also ignored. The incorporation of these parameters in future studies will be helpful in a proper identification of potential factors that contribute toward the development of anemia.

## Conclusions

The findings of this study with respect to anemia present a complex scenario that is controlled not only by the household economic status but also by education, diabetes, pregnancy, and nutritional status (BMI), which are associated as a causal factor of anemia. Therefore, the country should employ strategies to reduce diabetes and should consider in its policy strategy reducing the anemia during pregnancy, for example, the iron supplementation during pregnancy.

## Data Availability

The data that support the findings of this study are available from the DHS website (https://www.dhsprogram.com/) but restrictions apply to the availability of these data, which were used under license for the current study, and so are not publicly available. Data are however available upon reasonable request.

## Abbreviations

OR: Odd Ratio
CI: Confidence Interval
BMI: Body Max Index
SES: Socio Economic Status
NIPORT: National Institute of Population Research and Training
CKD: chronic kidney disease
BDHS: Bangladesh Demographic and Health Survey
